# Exploiting Dentine Matrix Proteins in Cell-Free Approaches for Periradicular Tissue Engineering

**DOI:** 10.1089/ten.teb.2021.0074

**Published:** 2022-08-08

**Authors:** Satnam Singh Virdee, Nasir Bashir, Josette Camilleri, Paul R. Cooper, Phillip L. Tomson

**Affiliations:** ^1^ Institute of Clinical Sciences, School of Dentistry & Birmingham Dental Hospital, University of Birmingham, Birmingham, United Kingdom.; ^2^ Department of Oral Sciences, Faculty of Dentistry, University of Otago, Dunedin, New Zealand.

**Keywords:** dentine extracellular matrix components, endodontics, regenerative medicine, stem cells, tissue regeneration, wound healing

## Abstract

The recent discovery of mesenchymal stem cells within periapical lesions (PL-MSC) has presented novel opportunities for managing periradicular diseases in adult teeth by way of enhancing tissue regeneration. This discovery coincides with the current paradigm shift toward biologically driven treatment strategies in endodontics, which have typically been reserved for non-vital immature permanent teeth. One such approach that shows promise is utilizing local endogenous non-collagenous dentine extracellular matrix components (dECM) to recruit and upregulate the intrinsic regenerative capacity of PL-MSCs *in situ*. At picogram levels, these morphogens have demonstrated tremendous ability to enhance the cellular activities in *in vitro* and *in vivo* animal studies that would otherwise be necessary for periradicular regeneration. Briefly, these include proliferation, viability, migration, differentiation, and mineralization. Therefore, topical application of dECMs during ortho- or retrograde root canal treatment could potentially enhance and sustain the regenerative mechanisms within diseased periapical tissues that are responsible for attaining favorable clinical and radiographic outcomes. This would provide many advantages when compared with conventional antimicrobial-only therapies for apical periodontitis (AP), which do not directly stimulate healing and have had stagnant success rates over the past five decades despite significant advances in operative techniques. The aim of this narrative review was to present the novel concept of exploiting endogenous dECMs as clinical tools for treating AP in mature permanent teeth. A large scope of literature was summarized to discuss the issues associated with conventional treatment modalities; current knowledge surrounding PL-MSCs; composition of the dECM; inductive potentials of dECM morphogens in other odontogenic stem cell niches; how treatment protocols can be adapted to take advantage of dECMs and PL-MSCs; and finally, the challenges currently impeding successful clinical translation alongside directions for future research.

**Impact statement:**

Apical periodontitis (AP) is an inflammatory condition that is associated with a great degree of morbidity and ultimately leads to tooth loss. The purpose of this review was to summarize the current evidence pertaining to stem cell therapy in endodontics and present a novel clinical methodology through which they may be utilized to address AP. A comprehensive overview of the basic science, clinical translation, and potential challenges are presented in this review.

## Introduction

Apical periodontitis (AP) is an inflammatory condition of the periodontium that exists when there is a dynamic equilibrium between putative endodontic microorganisms and host defense mechanisms.^1^ The ideal objective for treating this disease is to restore architecture and functions of the periradicular tissues that were lost to the immune response. Conventional therapies achieve these outcomes indirectly by reducing the microbial load within infected root canals to create a pro-healing environment.^2^ Although this approach may be enough to initiate periapical wound healing, which involves a highly co-ordinated sequence of hemostasis, inflammation, proliferation, and remodeling,^3^ it offers no additional stimulus for biological regeneration thereafter.^4^

Unaided, these endogenous processes are often insufficient to achieve complete tissue regeneration and will instead be compensated by reparative scar tissue.^4^ Persistent periapical radiolucencies may, therefore represent not only failure to eradicate intraradicular infection but also inadequate physiological regenerative processes, which could explain why larger lesions demonstrate higher treatment failure rates.^5,6^ It also suggests that to attain more predictable outcomes, it would be necessary to employ alternative strategies that simultaneously manage the microbial load and directly enhance intrinsic regenerative events within damaged periradicular tissues.

**FIG. 1.**
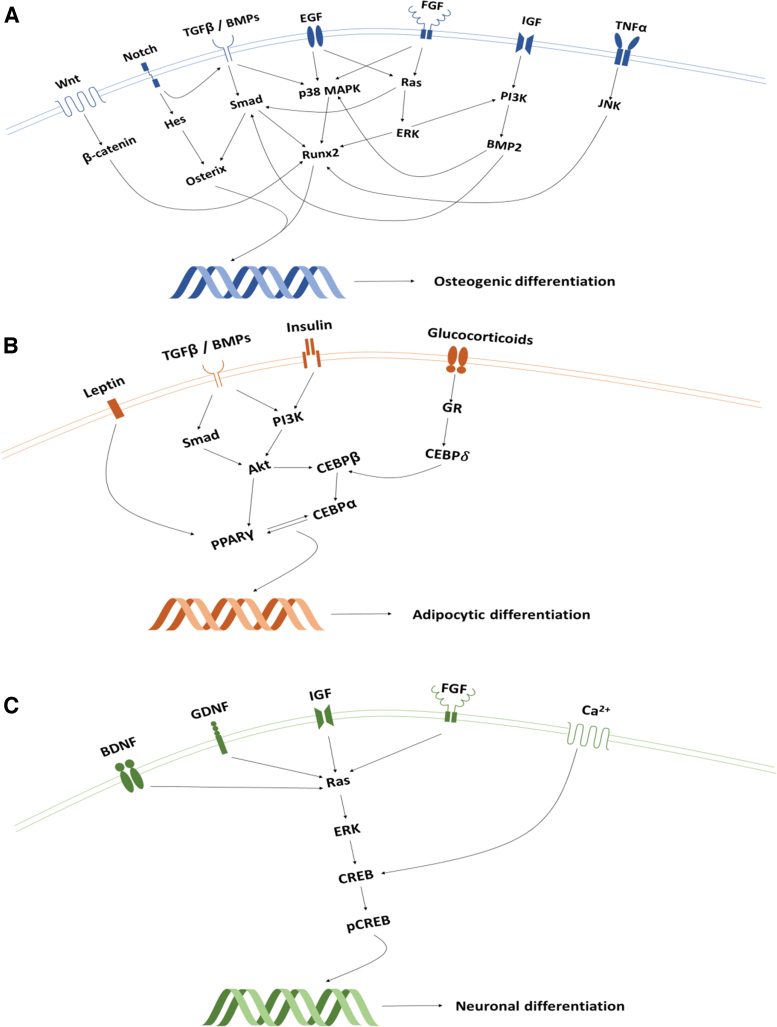
**(A–C)** A schematic illustration of osteogenic, adipocytic, and neuronal differentiation pathways in mesenchymal stem cells. Akt, protein kinase B; BDNF, brain-derived neurotrophic factor; BMP, bone matrix protein; Ca^2+^, calcium ions; CEBP, enhancer binding protein; CREB, cAMP response element-binding protein; EGF, epithelial growth factor; ERK, extracellular signal-regulated kinases; FGF, fibroblast growth factor; GDNF, glial cell line-derived neurotrophic factor; GR, glucocorticoid receptor; HES, hairy and enhancer of split-1; IGF, insulin-like growth factor; Jnk, c-Jun N-terminal kinases; MAPK, mitogen-activated protein kinase; PI3K, phosphoinositide 3-kinase; PPAR, peroxisome proliferator-activated receptor; Runx2, runt-related transcription factor 2; TGFβ, transforming growth factor beta; TNF-α, tumour necrosis factor-alpha; Wnt, wingless/integrated. Color images are available online.

Stem cells are essential to wound-healing processes, as they possess high proliferation rates, self-renewal capabilities, and potential for multi-lineage differentiation.^7,8^ Embryonic stem cells are pluripotent, as they can develop into stromal cells from any of the three germinal layers whereas multipotent postnatal stem cells are more restricted to organ-specific lineages.^9^ The latter are more amenable to clinical translation due to their autologous nature and presence within almost all adult tissues.^10^ A subset of multipotent progenitors derived from the mesoderm germ layer, called “mesenchymal stem cells” (MSC), has attracted particular interest within regenerative endodontics as they can give rise to several mineral producing mesoderm lineages, including bone (Fig. 1).^11^

Moreover; although they are known to be harvested from bone marrow, other reservoirs have been isolated from within the pulp and associated periodontal tissues of permanent and deciduous teeth.^9,12,13^ Named according to their tissue of origin, these “dental MSC” niches include “dental pulp stem cells” (DPSC), “stem cells from human exfoliated deciduous teeth” (SHED), “periodontal ligament stem cells” (PDLSC), “dental follicle precursor cells” (DFPC), “stem cells of the apical papilla” (SCAP), “gingival MSCs,” “alveolar bone MSCs,” and “tooth germ progenitor cells.”^9,12^ When transplanted into *in vivo* human and animal models, these dental MSCs have demonstrated a potent capacity to regenerate pulp-like tissue in empty root canals,^14–16^ dentine-like tissues in endodontic perforation defects,^17^ and periodontal tissues in surgically created periodontal defects.^18–20^

Further, the positive outcomes revealed from their applications to other non dento-alveolar tissues, including the treatment of autoimmune, cardiovascular, endocrine, hepatic, musculoskeletal, neurodegenerative, ophthalmic, dermatological, and respiratory diseases, confirm their potential to be utilized as powerful therapeutic tools (Supplementary Table S1). Recent studies, however, have identified another clinically accessible dental MSC population directly within the inflamed periradicular tissues of infected mature permanent teeth.^21,22^ These periapical lesion-derived MSCs (PL-MSC) possess tremendous immunosuppressive and regenerative potential and could, therefore, provide exciting opportunities to develop therapies for AP that actively engage with the endogenous mechanisms of periradicular tissue regeneration.

The cellular events required for periradicular regeneration are co-ordinated by various growth factors, cytokines, chemokines, and angiogenic and neurotrophic signaling molecules.^23^ Noteworthy examples include members of the transforming growth factor-beta (TGF-β), bone morphogenetic protein (BMP), fibroblast growth factor (FGF), vascular endothelial growth factor (VEGF), and insulin growth factor (IGF) families, among many others.^24^ Although these polypeptides are endogenously secreted by host cells at the site of disease, they rapidly deplete due to their relatively short half-life within the extracellular environment.^23^ Fortunately, abundant reservoirs of these molecules are locally sequestered within the dentine's extracellular matrix.^25^ They are deposited by secreting odontoblasts during dentinogenesis and become fossilized during subsequent mineralization.

Thereafter, their bioactivity remains highly preserved through the formation of proteoglycan bonds but these can be immediately reinstated on release.^26,27^ This has previously been achieved on command through demineralizing irrigants,^28–30^ pulp capping agents,^31–33^ epigenetic modifiers,^34^ and dental adhesives.^35^ The resulting extracts, formally termed “dentine extracellular matrix components” (dECM), have demonstrated a potent capacity to upregulate regenerative events within various odontogenic MSC niches.^33,36,37^ It is, therefore, plausible to expose PL-MSCs *in situ* to this cocktail of bioactive molecules to enhance local tissue healing. This approach could overcome current limitations associated with conventional treatments for AP and provide clinicians with unique capabilities to actively apply a biologically driven therapy to the diseased periradicular tissues.

The aim of this narrative review was to explore the novel concept of exploiting endogenous dECMs to upregulate local MSC-mediated periradicular tissue regeneration in mature permanent teeth diagnosed with AP. All abbreviations used in this article are provided in Table 1.

**Table 1.** Definitions of Abbreviations Found in Text

Abbreviation	Definition
AP	Apical periodontitis
BDNF	Brain-derived neurotrophic factor
BMP	Bone morphogenetic proteins
BSP	Bone sialoprotein
CD	Cluster of differentiation
CXCR4	Chemokine receptor type 4
dECM	Dentine extracellular matrix components
DFPC	Dental follicle precursor cells
DMP-1	Dentine matrix protein 1
DPP	Dentine phosphoprotein
DPSC	Dental pulp stem cells
DSPP	Dentin sialophosphoprotein
EDTA	Ethylenediaminetetraacetic acid
ESE	European Society of Endodontology
FGF	Fibroblast growth factors
HGF	Hepatocyte growth factor
IGF	Insulin growth factor
IL	Interleukin
MEPE	Matrix extracellular phosphoglycoprotein
MMP	Matrix metalloproteinases
MSC	Mesenchymal stem cells
NaOCl	Sodium hypochlorite
NGF	Nerve growth factor
NT3	Neurotrophin 3
NT4	Neurotrophin 4
OCN	Osteocalcin
ON	Osteonectin
OPN	Osteopontin
PDGF	Platelet-derived growth factor
PDLSC	Periodontal ligament stem cell
PlGF	Placental-derived growth factor
PL-MSC	Periapical lesion-derived mesenchymal stem cell
RUNX2/CBFA1	Runt-related transcription factor 2
SCAP	Stem cells of the apical papilla
SDF-1	Stromal-derived factor 1
SHED	Stem cells from human exfoliated deciduous teeth
TGF-β	Transforming growth factor-beta
TIMP	Tissue inhibitors of matrix metalloproteinases
TNF-α	Tumour necrosis factor alpha
VEGF	Vascular endothelial growth factors

## Current Issues Associated with Conventional Root Canal Therapy

Kakehashi *et al.* confirmed a direct causal relationship between putative endodontic microorganisms and periapical disease.^38^ Consequentially, therapeutic strategies for AP have focused exclusively on disinfecting necrotic root canals with the aim of relieving clinical signs and symptoms of inflammatory disease, preventing systemic bacterial spread, and ultimately retaining natural and functioning teeth.^2^ These outcomes are typically achieved through the use of antimicrobial solutions, primarily sodium hypochlorite (NaOCl), which possesses potent bactericidal and proteonacious properties, in conjunction with canal enlarging instruments.^39^

Significant advances in the chemo-mechanical debriding armamentarium have been made over the past 50 years, with some of the most revolutionary developments including highly flexible rotary/reciprocating file systems and machine-assisted irrigant agitation techniques. When compared with more conventional approaches, these now widely used practices facilitate deeper irrigant penetration into root dentine,^40^ greater intracanal debris and smear layer removal,^41^ and reductions in endodontic bacterial load and viability.^42,43^ It is, therefore, apparent that the operator's ability to disinfect root canals has significantly improved since the fundamental principles of endodontic therapy were first established.

Unfortunately, the aforementioned progress has not translated into improved clinical outcomes as success rates for root canal treatment have remained static for five decades. For instance, a systematic review by Ng *et al.* (2007) revealed that pooled success rates of all prior observational studies at 1 year follow-up ranged between 68% and 85% according to strict plain-film radiographic criteria.^44^ Thereafter, several prospective cohort studies reported comparable results of 83.0% (2–4 year follow-up) and 82.7% (5 year follow-up),^5,6^ with those reviewing patients over longer periods revealing even less favorable outcomes of 65.3% (20 year follow-up).^45^ Therefore, one out of five teeth with primary AP will in the short to medium term fail to heal after root canal treatment and eventually require more complicated and invasive remedial therapy.

Moreover, these figures likely underestimate the true incidence of treatment failure, as plain-film radiographs lack sensitivity for detecting periapical pathosis when compared with three-dimensional imaging techniques.^46,47^

Another issue is that microorganisms, and their by-products, cannot be completely eradicated from root canal systems due to complicated anatomy.^48^ Even root-filled teeth exhibiting no clinical or radiographic signs of AP harbor vital bacteria.^49,50^ Several inferences can be drawn from this finding. First, the ultimate objective of conventional approaches may be too idealistic, as residual bacteria are postoperatively unavoidable. Second, below a certain microbial load host mechanisms are capable of initiating, but not necessarily sustaining, regenerative events. Third, after surpassing this critical threshold, further disinfection provides no additional stimulus for endogenous periapical healing. These concepts are supported by several robust clinical investigations by Paredes-Vieyra *et al.*, Liang *et al.*, and Verma *et al.* who, respectively, demonstrated that intracanal medicaments, irrigant agitation techniques, and concentrated NaOCl solutions do not increase treatment success when compared with less aggressive disinfection protocols.^51–53^

It can be surmised that the effects of antimicrobial-only approaches on periradicular healing are finite and alternative methods, designed to initiate and sustain tissue healing, may yield more predictable outcomes. It must be stressed, however, that adequate endodontic disinfection still remains a fundamental prerequisite to provide an adequate micro-environment for any tissue repair strategy.

## Periapical Lesion-Derived MSCs

In 2004, Maeda *et al.* successfully isolated “fibroblastic cells” from within the inflamed periradicular granulation tissues of mature infected teeth.^54^ Thereafter, Liao *et al.*, Đokić *et al.*, and Marrelli *et al. in vitro* all confirmed their highly proliferative, multipotent, and clonogenic properties.^21,55,56^ Further, mesenchymal surface markers, Cluster of Differentiation [CD]-13, -29, -44, -73, -90, -105, and -166 were highly expressed; whereas hematopoietic markers, namely CD-14, -19, -34, -45, and human leukocyte antigen-DR isotype, were not.^21,55–61^ These characteristics fulfilled the minimum criteria necessary for this population to be recognized as a distinct MSC niche. Although many terms have been used to refer to this group, “PL-MSCs” is considered most accurate in the absence of explicit histological diagnoses and thus is the preferred designation (Table 2).

**Table 2.** Key Characteristics of Periapical Lesion-Derived Mesenchymal Stem Cells

Aliases	Immunophenotype		Proliferation		Differentiation markers			Mineralisation	Immunoregulatory effects
	Positive	Negative	Niche	Rate	Cell type	Genetic	Staining		
Granulation tissue-derived stem cells Human fibroblastic cells Human periapical cyst-derived mesenchymal stem cells Inflamed periapical progenitor cells Periapical lesion-derived stem cells	CD-13, CD-29, CD-44, CD-46, CD-73, CD-90, CD-105, CD-146, CD-166, Stro-1	CD-14, CD-19, CD-34, CD-45, HLA-DR	DFPC DPSC PDLSC PL-MSC SCAP SHED	++ ++ +++ + +++ ++	Odontoblast Osteoblast Cementoblast Chondrocyte Adipocyte Astrocyte	*DMP-1, DSSP* *ALP, BSP, MEPE, ON, OPN, RunX2/Cbfa1* *BSP, OCN, OPN* — *ADIPOQ, GLUT-4, LPL, PPARɣ* *DAT, En1, Foxa2, GFAP, MAP2, MSX1, NF-H, NF-M, Nurr1, Pitx3, TH, β-III tubulin*	Alizarin Red S Alizarin Red S Alizarin Red S Alcian Blue Oil Red O —	Calcific Tissues Fibrous Tissues	Increases leukocytic production of TGF-β Inhibits differentiation of dendritic cells Reduces leukocytic production of IL-1β, -2, -5, -6, TNF-α, and IFN-ɣ Reduces leukocytic proliferation

ADIPOQ, adiponectin; ALP, alkaline phosphatase; BSP, bone sialoprotein; CD, cluster of differentiation; DAT, dopamine transporter; DFPC, dental follicle precursor cells; DMP-1, dentine matrix protein 1; DPSC, dental pulp stem cell; DSSP, dentin sialophosphoprotein; En1, engrailed-1; Foxa2, forkhead box protein A2; GFAP, glial fibrillary acidic protein; GLUT-4, glucose transporter type 4; HLA-DR, human leukocyte antigen-DR isotype; IFN-ɣ, interferon gamma; IL, interleukin; LPL, lipoprotein lipase; MAP2, microtubule-associated protein 2; MEPE, matrix extracellular phosphoglycoprotein; MSX1, msh homeobox 1; NF-H, neurofilaments heavy; NF-M, neurofilaments medium; Nurr1, nuclear receptor related 1 protein; OCN, osteocalcin; ON, osteonectin; OPN, osteopontin; PDLSC, periodontal ligament stem cell; Pitx3, paired-like homeodomain transcription factor 3; PL-MSC, periapical lesion-derived stem cell; PPARɣ, peroxisome proliferator-activated receptor gamma; RunX2/Cbfa1, runt-related transcription factor 2; SCAP, stem cells of the apical papilla; SHED, stem cells from human exfoliated deciduous teeth; TGF-β, transforming growth factor-beta; TH, tyrosine hydroxylase; TNF-α, tumour necrosis factor alpha; +, low; ++, medium; +++, high.

**FIG. 2.**
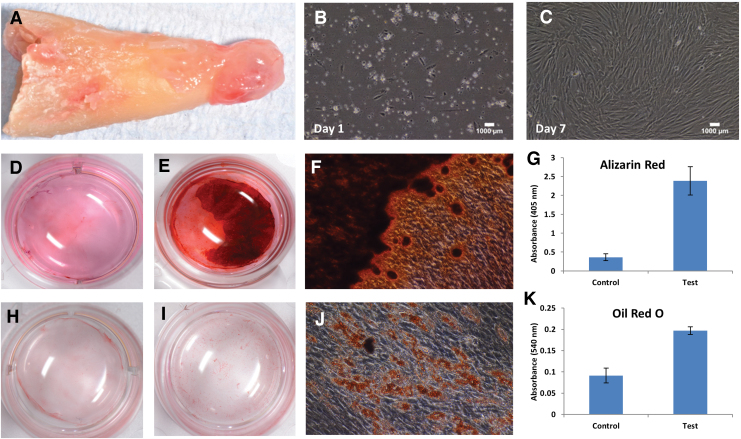
Multipotent potential of primary PL-MSCs. **(A)** PL-MSCs were isolated from the apical granuloma of extracted teeth via a collagenase type 1 enzyme digestion technique. Cells were cultured in a T25 flask with 20% fetal bovine serum supplemented α-MEM media, which was changed every 2 days. **(B, C)** Phase-contrast microscopy at 10 × magnification of PL-MSC cultures at day 1 **(B)** and day 7 **(C)**. **(D–G)** Osteogenic differentiation after 21 days of culture with control or osteogenic induction media (α-MEM, 20% FBS, 1% penicillin/streptomycin, 2 mM glutamine, 0.2 mM ascorbic acid, 100 nm dexamethasone, 10 mM β-glycerophosphate). Staining with Alizarin Red S confirmed absence in control wells **(D)** and presence in test wells of mineral deposits **(E, F)**. Staining was solubilised with 10% acetic acid, and subsequent intensity was quantified by using a microplate reader with an excitation wavelength set at 405 nm **(G)**. **(H–K)** Adipogenic differentiation after 21 days of culture with control or adipogenic induction media (α-MEM, 20% FBS, 1% penicillin/streptomycin, 2 mM glutamine, 0.5 mM IBMX, 200 μM indomethacin, 10 μM insulin, 1 μM dexamethasone). Staining with Oil Red O confirmed absence in control wells **(H)** and presence in test wells of lipid droplets **(I, J)**. Staining was solubilized with isopropanol, and subsequent intensity was quantified by using a microplate reader with an excitation wavelength set at 540 nm **(K)**. All experiments were conducted up to passage 2 by using three biological replicates. Scale bars represent 1000 μm. Color images are available online.

Figure 2 outlines preliminary data on the multipotent potential of primary PL-MSCs cultured from the apical granulomas of extracted teeth diagnosed with AP.

Clinical observations of periradicular regeneration after endodontic therapy indicate that PL-MSCs primarily contribute to local intrinsic periapical wound-healing processes. This is supported by *in vitro* investigations confirming that these cells possess the necessary capabilities to restore such tissues. For instance, with appropriate cues PL-MSCs differentiate into osteoblasts, cementoblasts, adipocytes, astrocytes, and chondrocytes, all of which are relevant for regenerating the periodontium.^54–56,58^

In addition, when compared with other odontogenic MSCs, these multipotent properties are more directed toward osteogenesis.^55,62^ This was demonstrated through gene expression analyses where upon osteogenic induction, PL-MSCs exhibited transcriptional profiles more indicative of osteogenic differentiation than DPSCs (osteonectin [*ON*], bone sialoprotein [*BSP*], runt-related-transcription-factor 2 [*RUNX2/CBFA1*]), which instead greatly expressed odontogenic markers (dentin sialophosphoprotein [*DSPP*], dentine matrix protein [*DMP*]-1).^62^ The mineralization needed for these cells to be considered functional has also been confirmed through several *in vitro* differentiation assays,^55,56,60^ as well as *in vivo* subcutaneous implantation mouse models.^21^

Such stem-like characteristics, however, do vary with CD146-positive PL-MSC subpopulations exhibiting lower proliferative, clonogenic, and osteogenic potential than CD146-negative subpopulations.^59^ These properties may also be dampened by the inflammatory microenvironment, as indicated by weaker proliferation rates when compared with healthy DPSCs and PDLSCs.^21,55,63^

Stem cells from periradicular lesions also possess immunomodulatory properties. For instance, Đokić *et al.* initially demonstrated that PL-MSC co-cultures significantly reduced leukocytic proliferation, differentiation, and pro-osteoclastic cytokine production (Interleukin [IL]-1β, -2, -5, -6, tumor necrosis factor [TNF]-α, Interferon-ɣ), while simultaneously increasing anti-inflammatory growth factor secretion (TGF-β).^55,57^ These results were corroborated by Araujo-Pires *et al.*, who *in vivo* detected a converse immunological profile in Chemokine Receptor Type [CXCR]4 knockout mice and higher expression of transcriptional markers for MSC mobilization (CD-29, -44, -73, CXCR4), differentiation (NANOG, Stro-1), and transmigration (CD-106, -166) within chronic, as opposed to acute, human periapical granulomas.^22^

More recently, Estrela *et al.* also observed a higher presence of MSCs within stable periradicular lesions. Collectively, these findings suggest that the immunosuppressive properties of PL-MSCs actively contribute to arresting progression of periapical diseases.^64^

Overall, the study investigations described earlier highlight the tremendous regenerative and immunomodulatory capabilities of PL-MSCs. They lay a strong foundation for preclinical *in vivo* studies, which should be performed, that explore their therapeutic potentials. Dentoalveolar, neurodegenerative, and skeletal diseases may particularly benefit from advances in this area due to the enhanced neurogenic and osteogenic commitment of this niche.^58,61,62,65^ Moreover, the immunomodulatory and mineralized regenerative properties demonstrated by PL-MSCs *in vitro* and *in vivo*, respectively, indicate that these cells are modulators of the periapical lesion healing process and thus making them ideal targets in novel tissue regeneration strategies for AP.^21^ One such approach would involve enhancing their regenerative capacity *in situ* by liberating endogenous signaling molecules from within the dentine's extracellular matrix.

## dECM Components

More than 280 bioactive molecules have been identified within demineralized dentine.^66,67^ A vast majority of these are non-collagenous extracellular matrix proteins,^66^ which comprise ∼10% of the dentine's organic phase and are considered crucial for dentinogenesis.^68^ Growth factors constitute large proportions of this cohort and have been implicated in regulating dentine-pulp reparative and regenerative responses. Members of the TGF-β, BMP, VEGF, FGF, IGF, platelet-derived growth factor (PDGF), hepatocyte growth factor (HGF), placental-derived growth factor (PlGF), epidermal growth factor, and adrenomedullin families are frequently detected, with TGF-β1 often found in the greatest abundance.^28,29,33,34,69–71^

Several of these, namely VEGFs, FGFs, PDGFs, and PlGFs, are also known mediators of angiogenesis, which is a critical wound-healing process involving the formation of new blood vessels.^72,73^ Closely associated with these are neurotrophic factors that are responsible for developing intricate innervations within the dentin-pulp complex.^74^ Isolated examples include nerve growth factor (NGF), brain-derived neurotrophic factor (BDNF), neurotrophin 3 and 4 (NT3/NT4), and glial cell–line derived neurotrophic factor.^75^ Further, a broad range of pro- and anti-inflammatory cytokines, namely IL-1α, -1β, -4, -6, -8, -10, -12, and granulocyte-macrophage colony-stimulating factor, have also been detected within solubilized dECMs.^32,76^ These NF-ĸB signaling molecules likely contribute to immunoregulatory pulp mechanisms, as indicated by their capacity to induce a wide array of inflammatory events.^77^

Other non-collagenous protein families released from the dentine matrix are those associated with regulating mineralization and maturation processes of human calcified tissues.^78^ Briefly, these include small integrin-binding ligand n-linked glycoproteins (*DMP-1*, *BSP*, osteopontin [*OPN*], dentine phosphoprotein [*DPP*], dentine sialoprotein, dentine glycoprotein, matrix-extracellular-phosphoglycoprotein [*MEPE*]); vitamin K-dependent glycoproteins (osteocalcin [*OCN*]); small leukine-rich proteoglycans (decorin, biglycan, fibromodulin, lumican, osteoadherin); secretory calcium-binding phosphoproteins (*ON*); and large aggregating proteoglycans (versican).

Many of these require enzymatic activation and therefore it is not unexpected that the dentine substrate also contains matrix metalloproteinases ([MMP]-2, -3, -8, -9, -20) and tissue inhibitors of MMPs ([TIMP]-1,-2),^79–82^ which also regulate extracellular matrix remodeling. Although serum proteins (albumin, Immunoglobulin-A, -M, Transferin, Fetuin-A) are also present, currently their functions are unknown.^83,84^

**FIG. 3.**
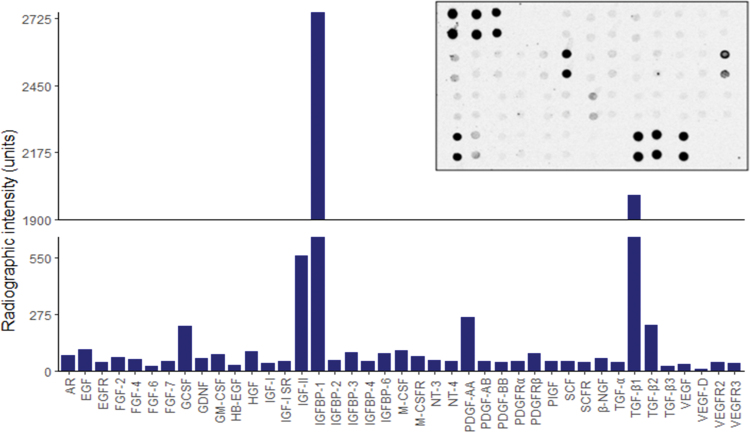
Human growth factor anti-body array of lyophilized dECM components extracted from dentine powder using 10% EDTA. A semi-quantitative autoradiographic image analysis technique was used to determine the relative radiographic intensity for a total of 41 different cytokines. A representative autoradiographic image is displayed in the top left corner. AR, amphiregulin; EGFR, epidermal growth factor receptor; GCSF, granulocyte colony-stimulating factor; GM-CSF, granulocyte macrophage colony-stimulating factor; HB-EGF, heparin-binding epidermal growth factor; HGF, hepatocyte growth factor; IGFBP, insulin-like growth factor binding protein; M-CSF, macrophage colony-stimulating factor; M-CSFR, macrophage colony-stimulating factor receptor; NGF, nerve growth factor; NT, neurotrophin; PDGF, platelet-derived growth factor; PDGFR, platelet-derived growth factor receptor; PlGF, placental growth factor; SCF, stem cell factor; SCFR, stem cell factor receptor; VEGF, vascular endothelial growth factor; VEGFR, vascular endothelial growth factor receptor. Color images are available online.

Given what has been cited earlier, dentine can no longer be considered an inert structural tissue but instead, a reservoir of potentially exploitable therapeutic auto- and paracrine cell-signaling molecules that resembles other connective tissues such as bone.^85^ Figure 3 represents the results of a broad human anti-body array conducted by our own research group on lyophilized dECM components extracted from dentine powder using ethylenediaminetetraacetic acid (EDTA). The endogenous nature of these morphogens overcomes many ethical issues associated with clinically using exogenous substitutes and the synergistic activity within solubilized dECMs; it exhibits a greater potency than single recombinant molecules.^37,86,87^ For these reasons, dECM extracts have been extensively studied for their ability to initiate regenerative events within various oral and dental MSC niches.

## Effects of dECM Components on Dental Stem Cells

The effects of dECM application on dental MSC niches are summarized in Table 3.

**Table 3.** Regenerative Effects of Dentine Extracellular Matrix Component on Odontogenic Stem Cell Niches

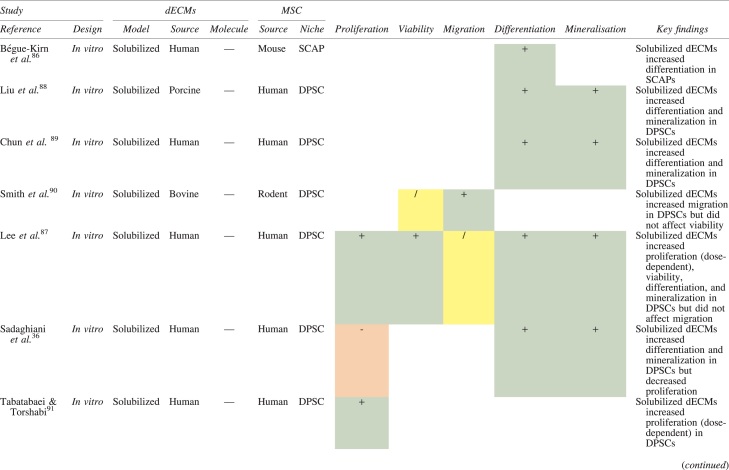 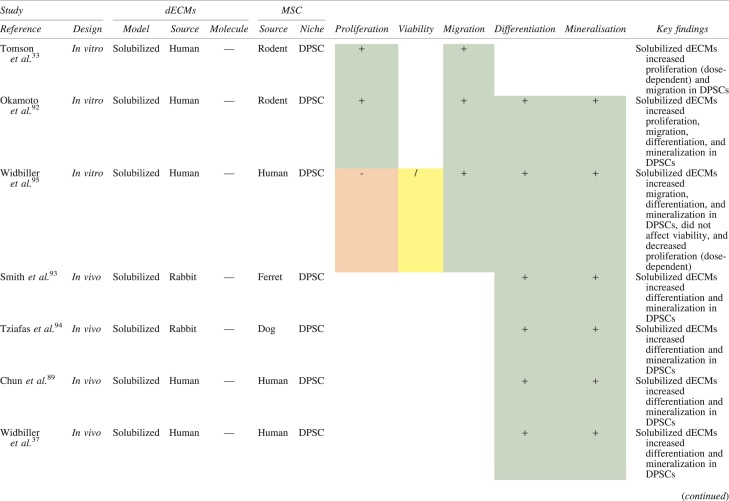 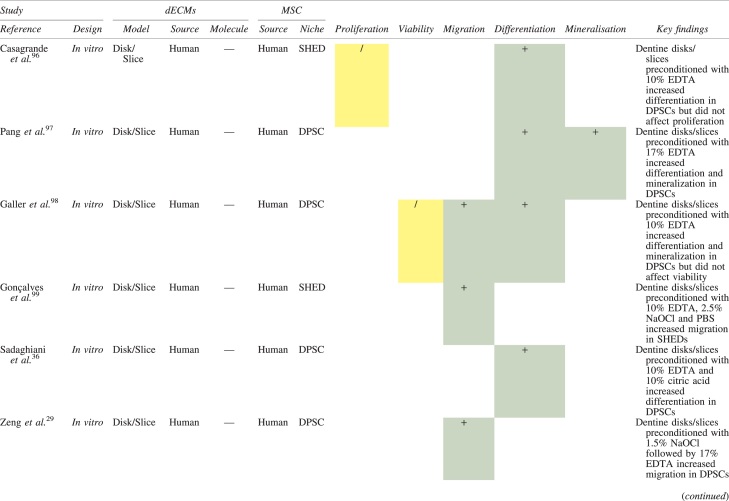 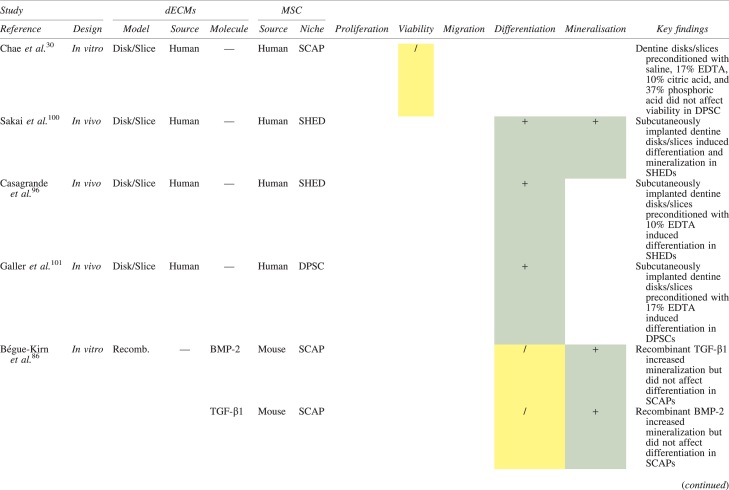 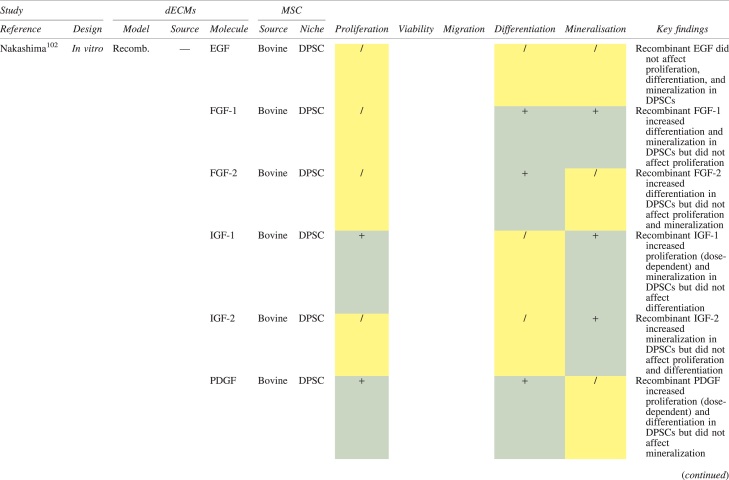 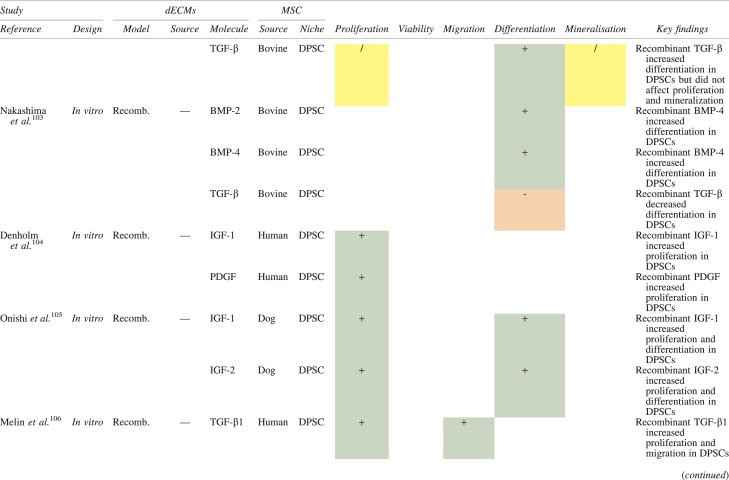 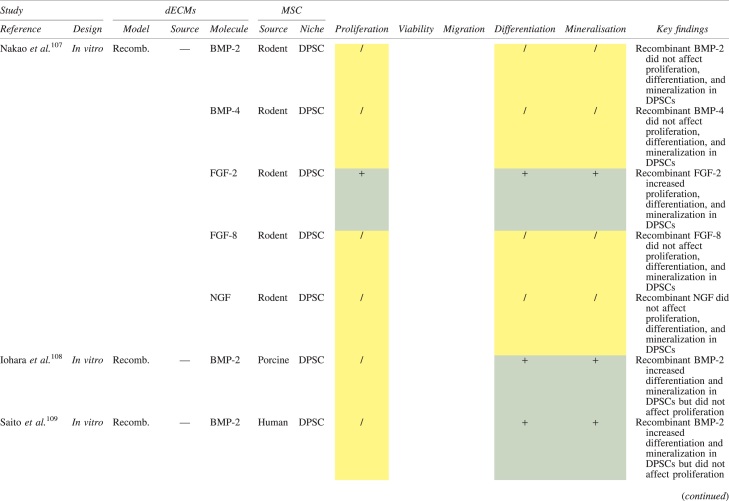 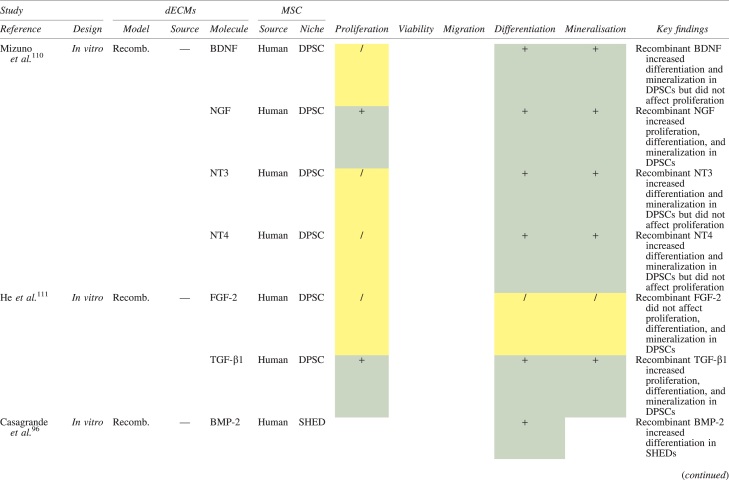 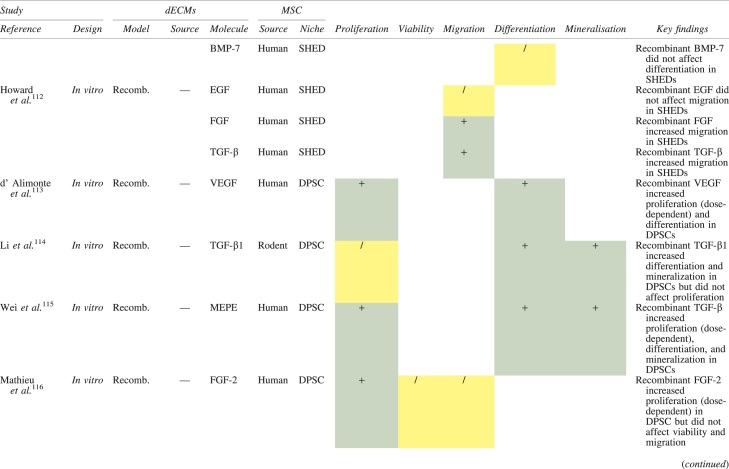 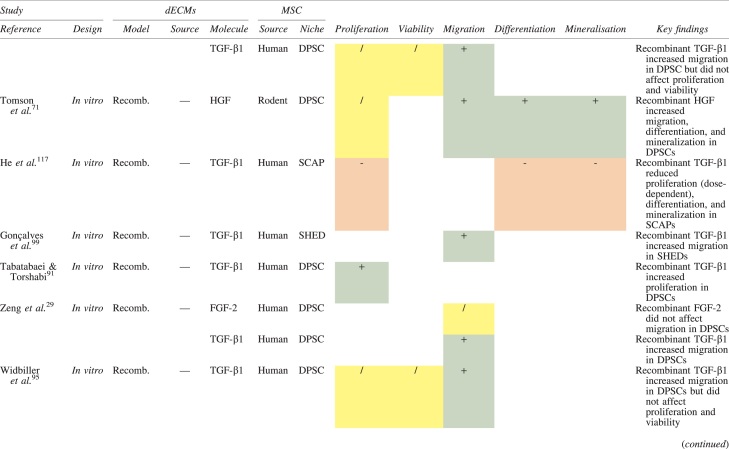 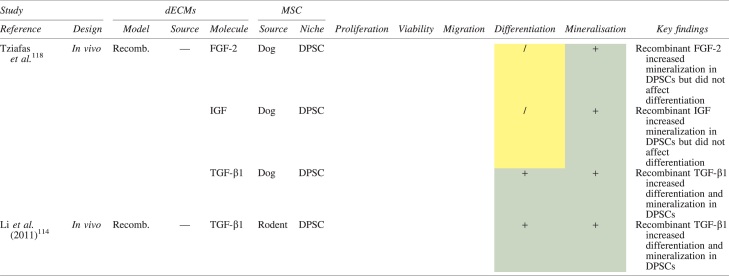

Studies have been arranged primarily on the dECM model used, followed by study design and then date.

BDNF, brain-derived neurotrophic factor; BMP, bone morphogenetic protein; dECM, dentin extracellular matrix component; DPSC, dental pulp stem cell; EGF, epidermal growth factor; FGF, fibroblast growth factor; HGF, hepatocyte growth factor; IGF, insulin growth factor; MEPE, matrix extracellular phosphoglycoprotein; MSC, mesenchymal stem cell; NGF, nerve growth factor; NT3, neurotrophin 3; NT4, neurotrophin 4; PDGF, platelet-derived growth factor; Recomb, recombinant; SCAP, stem cells of the apical papilla; SHED, stem cells from human exfoliated deciduous teeth; TGF-β, transforming growth factor beta; VEGF, vascular endothelial growth factor; +, increased; -,decreased; /, nil. Color images are available online.

### Migration

Dentine matrix components have demonstrated chemotactic properties *in vitro* via transwell migration, matrigel invasion, and scratch wound assays.^33,90,92^ When solubilized, these extracts exhibit considerable potency with DPSC recruitment occurring at just picogram levels.^37^ Moreover, root segments pre-conditioned with demineralizing agents induce similar migratory effects in DPSCs and SHEDs, which contrasts the relatively inert properties of their deproteinized counterparts.^29,98,99^ Other *in vitro* studies using single recombinant growth factors indicate that these properties can be attributed to the presence of known chemoattractants such as TGF-β1, HGF, and FGF.^29,71,106,112,116^

### Proliferation

Solubilized dECMs induce time- and dose-dependent MSC proliferation. These properties, however, are observed only up to a critical threshold, after which anti-mitogenic events become apparent. For example, dECM applications less than 100 μgmL^−1^ enhance DPSC proliferation *in vitro*,^33,87,91,92^ whereas greater concentrations inhibit further growth.^36,37^ This observation, which is also witnessed in endothelial cell cultures and angiogenic tube formation assays,^73^ could be explained as being the net outcome induced by various molecules within dECM extracts. Some constituents, namely TGF-β1,^117,119^ inhibit proliferation in several cell types but may also attenuate effects of other stimulatory growth factors such as MEPE, PDGF, VEGF, IGF, and FGF.^102,104,105,107,113,115,116^ Moreover, dECM-induced terminal differentiation may further contribute to reducing cell numbers over time.^36^

### Apoptosis

Solubilized extracts induce limited apoptotic effects in MSCs.^37,87,90,98^ Higher dECM concentrations have even been found to aid DPSC viability, as indicated by reduced caspase-3 activity and increased serine threonine kinase gene expression.^87^ This could be accredited to dentinal morphogens that possess anti-apoptotic potential such as DPPs and PDGF, which activate downstream signaling cascades for cell survival.^120,121^

### Differentiation

Numerous *in vitro* studies using DPSCs, SCAPs, and SHEDs indicate that dECM extracts are powerful inducers of osteo- and odontoblastic differentiation. For instance, topical applications stimulate organization and formation of elongated cellular processes that extend into tubules of pre-conditioned dentine disks.^88,89,97,98^ This is accompanied by significant increases in mRNA expression for genes characteristic of odonto- and osteogenic commitment. These include *DSPP*, *DMP-1*, *OPN*, *OCN*, *BSP*, *RUNX2/CBFA1*, *MEPE*, type 1 collagen, alkaline phosphatase, distal-less homeobox 5, and msh homeobox 2.^36,37,87,92,96,97^

In addition, when DPSCs and SHEDs are implanted subcutaneously alongside dECMs, differentiation events still transpire.^93–95,100,101,118^ Dentine-derived BMP-2, in particular, is essential in this process, as is demonstrated when blockade of BMP-2 signals, which are otherwise transduced down osteogenic smad-1/5/8 and *p*38 mitogen-activated-protein-kinase pathways, inhibited odontoblastic gene expression in SHEDs.^96^ Further, many studies using recombinant growth factors continue to display the potent differentiating activity of BMP-2.^103,107–109^ Nevertheless, other dentine morphogens that may act concomitantly include TGF-β1, although it exhibits suppressive effects via smad-3 dependent mechanisms in SCAPs; PDGF; FGF; BMP-4; IGF; HGF; VEGF; NGF; BDNF; NT3; NT4; MEPE; and TNF-α.^71,102,103,105,107,110,111,113–115,122^

### Mineralization

Colorimetric methods for calcium quantification demonstrate that dECMs significantly accelerate mineralised matrix production within MSCs.^37,84,87,88,92^ Calcified nodules indicating functioning osteo- and odontoblasts can be visually observed as early as 5 days post-exposure and become more prominent thereafter.^87^ When tested *in vivo,* using subcutaneous implantation models*,* this deposition leads to osseous, dentinal, and collagenous-like tissue formation.^93–95,100,114^ This feature can be ascribed to the ability of dentinal morphogens to upregulate genes that code for extracellular matrix protein production in teeth and bone.

Overall, dECMs possess bioactive properties that, if applied to PL-MSCs, could be of clinical utility for periradicular tissue regeneration.

## Potential Therapeutic Approach

**FIG. 4.**
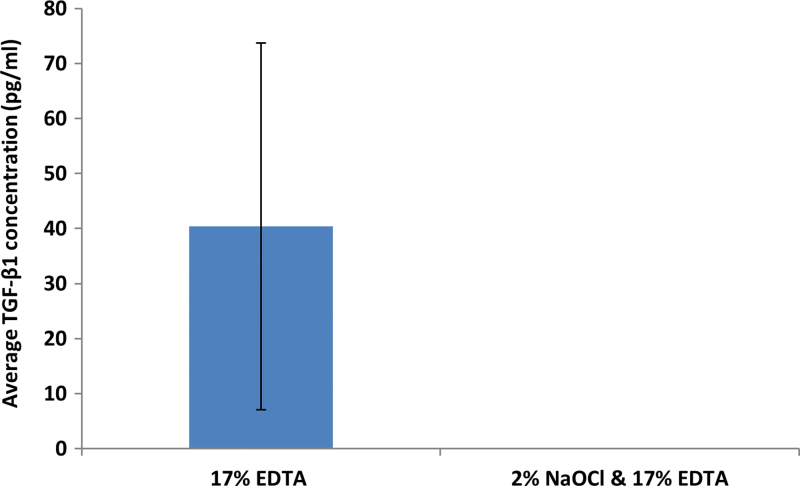
Data obtained from our research group demonstrating the deleterious effects of sodium hypochlorite on the solubilization of dentine extracellular matrix components when delivered into prepared root canals of extracted mature permanent human teeth (*n* = 10) via conventional needle irrigation. A sandwich ELISA technique was used to detect TGF-β1 concentration (pg/mL) from 100 μL of EDTA after irrigation with or without 2% sodium hypochlorite. Error bars represent standard deviation. Color images are available online.

The principles underlying cell-free homing techniques, where MSCs are recruited and stimulated *in situ* by supplying damaged tissues with signaling molecules,^13^ could be utilized to exploit endogenous dECMs for the treatment of AP in mature permanent teeth. Conceptually speaking, common chelating agents, namely, 17% EDTA, can be used as the primary irrigant throughout chemo-mechanical debridement to preserve and maximize the release of dentine matrix proteins into root canals. These would otherwise be negatively impacted by the proteonacious properties exhibited by even low concentrations of NaOCl.^28,99^ This observation is consistent with pilot data obtained by our own research group (Fig. 4).

Ultrasonic agitation has been found to significantly assist dECM release and, thus, is an essential irrigant adjunct after instrumentation.^123^ These solubilized morphogens could then be encouraged to egress into periapical tissues, by way of manual dynamic activation and patency filing. Subsequently, they would act as chemoattractants to local tissue PL-MSCs present within the peripheral capsular region of granulomas, and subsequently enhance their regenerative potential.^124^ This interaction will likely require precise pre-enlargement of the apical foramen after accurately determining its position.^13^

**FIG. 5.**
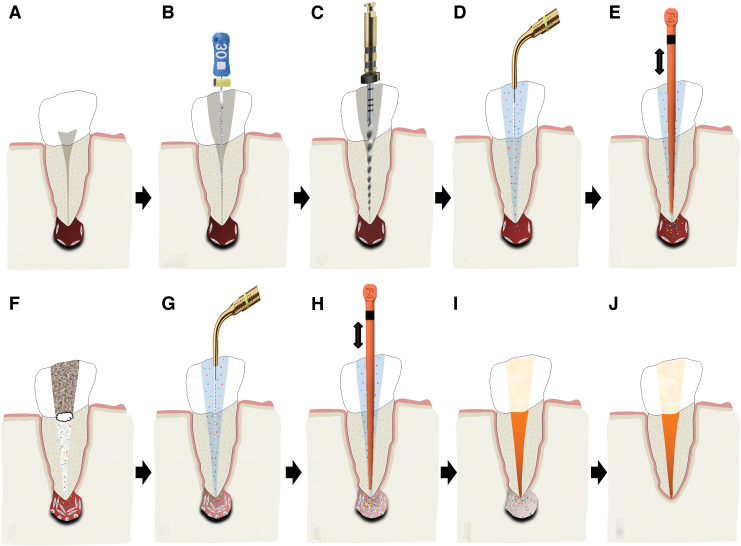
A schematic illustration of the proposed protocol for enhancing periradicular tissue regeneration in mature permanent teeth by using endogenous dECM components. **(A)** Single-rooted mature permanent tooth diagnosed with apical periodontitis; **(B)** accessing pulp chamber and conservative pre-enlargement of apical foramen; **(C)** chemomechanical preparation of root canal using a chelating agent; **(D)** passive ultrasonic activation of irrigant to stimulate release of dECMs into the root canal; **(E)** manual dynamic activation to encourage periapical bioavailability of dECMs; **(F)** interappointment calcium hydroxide medicament; **(G)** irrigation and passive ultrasonic activation to release dECMs; **(H)** manual dynamic activation to encourage periapical bioavailability of dECMs; **(I)** obturation; **(J)** annual clinical and radiographic review. Color images are available online.

Moreover; antimicrobial inter-appointment medicaments, namely calcium hydroxide, may further prolong dECM exposure due to their ability to liberate bioactive dentine molecules.^31,33^ This two-stage approach provides additional disinfection, potentially compensating for the absence of NaOCl, and helps confirm resolution of active disease before obturation. Thereafter, routine clinical and radiographic examination would be required to monitor periapical healing (Fig. 5).

The theoretical basis of the proposed approach is derived from preclinical animal studies that have utilized recombinant components of the dentine matrix to regenerate dentoalveolar tissues. For instance; Kim *et al.* reported that BMP-7 and stromal-derived factor-1 (SDF-1), delivered subcutaneously into rats via 200 μm micro-channels in bioprinted human molar scaffolds, increased both recruitment of endogenous MSCs and angiogenesis and ultimately led to regeneration of an anatomically shaped tooth like-structure.^125^

Remarkably, in this model a *de novo* periodontal ligament and alveolar bone was also observed as integrating with the native bone at the scaffold interface after 9 weeks. When the same molecules, plus FGF, were used to coat the roots of intentionally avulsed mandibular premolars in beagle dogs, they were found to contribute to the re-establishment of highly organized periodontal ligament tissues after delayed re-implantation.^126,127^ These neo-fibers inserted deeply into the adjacent cementum and alveolar bone and prevented the onset of external replacement or inflammatory root resorption.

Kim *et al.* were also able to demonstrate that without the use of stem cell transplantation, re-cellularized and re-vascularized dental pulp-like tissue was regenerated across the entire length of endodontically treated human-sized root canals after 3 weeks of exposure to FGF, VEGF, PDGF, NGF, and BMP7 in a subcutaneous implantation mouse model.^128^ Similar observations were reported by Suzuki *et al.*^129^ These studies, in particular, provide the strongest support for the proposed protocol as they demonstrate *in vivo* regeneration of the very tissues necessary for a *de novo* periodontium using a cell-free approach.

Further support for the homing potential of dECMs, however, comes from the applications of other prevalent dentine matrix proteins, such as DPP and DMP-1, in rat models. For example, exposure to DPP induced odontoblastic differentiation and subsequent reparative dentine bridge formation in inflamed pulp tissue and DMP-1 impregnated scaffolds exhibited marked extracellular matrix deposition and neovascularization in endodontic perforation defects.^17,130^

In other areas of medicine, stem cell homing techniques utilizing TGF-β3 molecules have successfully contributed to regenerating entire humeral condyles in rabbits after radical resection.^131^ Collectively, these findings have to date been clinically translated into novel pulp preservation and regeneration protocols and provide proof of concept for the therapeutic potentials of dECMs when used in stem cell homing techniques as described earlier.^132–134^ Nevertheless, although the proposed approach circumvents many ethical issues related to cell-based transplantation strategies, it is at present only speculative. Numerous hurdles are still required to be overcome before successful clinical translation.

## Challenges to Successful Clinical Translation and Directions for Future Research

The greatest challenge associated with implementing the protocol cited earlier is developing chemo-mechanical debridement regimes that sufficiently disinfect root canals while preserving dECMs and PL-MSCs. This would particularly affect NaOCl use, which has proven detrimental to stem cell viability,^135,136^ dentine matrix growth factor bioavailability,^28,99^ and induction of key tissue regeneration events.^96,101^ Although lower concentrations and contact times of 1.5% and 5 min, respectively, have been advocated for regenerative endodontic treatments, these parameters are derived from studies only investigating MSC viability.^98,135,136^ Therefore, it is currently unknown how they influence dECM release. Further, the ideal strength for NaOCl's antimicrobial efficacy is reported as 2.5%,^137^ which is otherwise cytotoxic to MSCs and significantly reduces the bioavailability of dECMs.^99,136^

What has been cited earlier suggests that if NaOCl were to be administered even in a limited capacity, its deleterious effects on dECMs would need mitigating, which is supported by pilot data (Fig. 4). This could perhaps be achieved by enhancing the activity of demineralizing agents or mechanically removing the affected dentinal substrate, the latter of which requires a prerequisite understanding of NaOCl's penetrative capabilities. However, should these methods lead to no avail, NaOCl will need to be substituted for alternative antimicrobial strategies. For instance, the thicker and less fragile root canal walls in mature permanent teeth allow for more emphasis on conventional instrumentation and intracanal medicaments, which have *in vivo* shown greater contribution to endodontic disinfection than lower NaOCl concentrations.^138^

Moreover; EDTA, which is currently considered a weak antimicrobial agent, destabilizes the outer cell membranes of gram-negative bacteria and deteriorates the macrostructures of established biofilms.^139^ Although these effects alone may not always induce cell death, they could potentially be enhanced enough to do so when combined with mechanical instrumentation and irrigant agitation techniques. The reductions in microbial load achieved through these mechanisms may equate to that of NaOCl treatment and exceed the threshold necessary to control infection while preserving the biological components within dentine.^2^ Further investigations are required to test these hypotheses.

Another challenge is that the effectiveness of the proposed strategy has yet to be proven in concept. Although the regenerative potentials of dECMs have been demonstrated in DPSCs, SCAPs, and SHEDs; it is currently unknown whether similar effects are observed in cultures of PL-MSCs. This niche has already demonstrated different stem-like characteristics and thus may yield results at variance to that of other MSCs.^55^ Animal studies, utilizing the intentional pulp exposure model of AP, could be employed to further support or challenge the aforementioned hypothesis. They would provide valuable histological and radiographic insight into the periradicular healing process at key time points after dECM exposure, which is data that ethically cannot be attained *in vivo* using human participants.

Rodents such as rats and mice provide researchers endodontic anatomy (i.e., molar teeth), infected root canal microflora, and wound-healing physiology comparable to that of humans and they conform to the public opposition of using larger animals, thus making them the species of choice.^140–142^ Overall, these preliminary studies are necessary to justify more time-consuming, labor-intensive, expensive, and appropriately powered prospective randomized controlled trials, which would be the ultimate means of demonstrating the clinical effectiveness of the proposed intervention. Such investigations would also benefit from more sensitive outcome measures that could longitudinally detect biological changes within the periradicular tissues.

## Conclusion

The discovery of multipotent stem cells within periapical lesions presents novel opportunities for managing AP by way of harnessing local tissue regeneration. Multiple *in vitro* studies have confirmed their immunomodulatory and stem cell-like characteristics, which implicates them as being key determiners of the periapical healing process and provides the foundations for subsequent *in vivo* investigation. Further, there is extensive evidence demonstrating that components within the dentine's extracellular matrix are capable of upregulating the very regenerative responses within dental MSCs that would otherwise be necessary for periradicular regeneration. This includes the enhancement of cellular proliferation, migration, viability, differentiation, and mineralization.

It is well established that these bioactive properties can be harnessed by clinicians on command with common chelating agents such as EDTA, which provides the theoretical and clinical basis of the proposed protocol. Further *in vitro* and *in vivo* studies, however, are still required to determine the regenerative effects of dECMs in PL-MSC cultures, optimal irrigant regimes for liberating dECMs, and their effects on the clinical success rates of root canal treatment. Such investigations at the very least would improve understanding of the biological mechanisms associated with periradicular healing, which could in future lead to the development of regenerative endodontic treatment strategies for AP.

## Supplementary Material

Supplemental data
