# Supplementary material for: Exploiting Dentine Matrix Proteins in Cell-Free Approaches for Periradicular Tissue Engineering
Source: Tissue Eng Part B Rev. 2022 Aug 8;28(4):707–32. doi: 10.1089/ten.teb.2021.0074 (PMC9419954; doi:10.1089/ten.teb.2021.0074)
Supplement: Supplemental data [file Suppl_TableS1.docx]

| Supplementary Table S1: *In vivo* studies evaluating the therapeutic potentials of of dental mesenchymal stem cells. Studies have been grouped based on primary disease treated. The references associated with this table are listed below. | | | | | |
| --- | --- | --- | --- | --- | --- |
| Study | Target Disease | | Niche | Animal | Key finding |
| Yamaza *et al.* (2010)^1^ | | Autoimmune | SHED | Mouse | SHED transplantation reversed systemic lupus erythematous associated disorders |
| Ishikawa *et al.* (2016)^2^ | | Autoimmune | SHED | Mouse | SHED-conditioned media infusion prevented exacerbation of rheumatoid arthritis symptoms |
| Shimojima *et al.* (2016)^3^ | | Autoimmune | SHED | Mouse | SHED-conditioned media infusion reduced neuronal injury associated with autoimmune encephalomyelitis |
| Gandia *et al.* (2008)^4^ | | Cardiovascular | DPSC | Rat | DPSC injection improved cardiac function after myocardial infarction |
| Yamaguchi *et al.* (2015)^5^ | | Cardiovascular | SHED | Rat | SHED-conditioned media infusion reduced infarct size after myocardial infarction |
| Chiu *et al.* (2016)^6^ | | Cardiovascular | DPSC | Rat | DPSC implantation promoted neurogenesis and angiogenesis in the presence of hypoxia-ischemia |
| Lee *et al.* (2016)^7^ | | Cardiovascular | DPSC | Rat | DPSC transplantation induced angiogenesis and improved neurological function after stroke |
| Miura *et al.* (2003)^8^ | | Dento-alveolar | SHED | Mouse | SHED transplantation increased dentine tissue-like deposition |
| Seo *et al.* (2004)^9^ | | Dento-alveolar | PDLSC | Mouse | PDLSC transplantation increased periodontal tissue regeneration in surgically created periodontal defects |
| Sonoyama *et al.* (2006*)^10^* | | Dento-alveolar | PDLSC / SCAP | Mouse | PDLSC / SCAP transplantation increased root/periodontal complex regeneration capable of supporting porcelain crowns |
| Liu *et al.* (2008)^11^ | | Dento-alveolar | PDLSC | Swine | PDLSC transplantation increased periodontal tissue regeneration in surgically created periodontal defects |
| Cordeiro *et al.* (2008)^12^ | | Dento-alveolar | SHED | Mouse | SHED transplantation improved regeneration of pulp-like tissue in tooth slices |
| Ding *et al.* (2010)^13^ | | Dento-alveolar | PDLSC | Swine | PDLSC transplantation increased periodontal tissue regeneration in surgically created periodontal defects |
| Huang *et al.* (2010)^14^ | | Dento-alveolar | DPSC / SCAP | Mouse | DPSC and SCAP seeding increased regeneration of vascularised pulp-like tissue in emptied root canals |
| Alsanea *et al.* (2011)^15^ | | Dento-alveolar | DPSC | Mouse | DPSC transplantation increased dentine regeneration in endodontic perforation defects |
| Park *et al.* (2011)^16^ | | Dento-alveolar | DPSC / PDLSC | Canine | PDLSC transplantation increased periodontal tissue regeneration in surgically created periodontal defects |
| Khorsand *et al.* (2013)^17^ | | Dento-alveolar | DPSC | Canine | PDLSC transplantation increased periodontal tissue regeneration in surgically created periodontal defects |
| Rosa *et al.* (2013)^18^ | | Dento-alveolar | SHED | Mouse | SHED injection increased regeneration of vascularised pulp-like tissue in emptied root canals |
| Yu *et al.* (2013)^19^ | | Dento-alveolar | GMSC | Canine | GMSC transplantation increased periodontal tissue regeneration in surgically created periodontal defects |
| Nakashima & Iohara (2014)^20^ | | Dento-alveolar | DPSC | Canine | DPSC seeding increased regeneration of vascularised pulp-like tissue in emptied root canals |
| Kanafi *et al.* (2013)^21^ | | Endocrine | SHED | Mouse | SHED-derived islet cell transplantation reversed streptozotocin-induced diabetes |
| Cho *et al.* (2015)^22^ | | Hepatic | DPSC | Mouse | DPSC transplantation suppressed liver fibrosis and improved liver function |
| Yamaza *et al.* (2015)^23^ | | Hepatic | SHED | Mouse | SHED transplantation improved liver dysfunction and reduced fibrosis |
| de Mendonça Costa  *et al.* (2008)^24^ | | Musculoskeletal | DPSC | Rat | DPSC transplantation increased bone regeneration in cranial defects |
| d’Aquino *et al.* (2009)^25^ | | Musculoskeletal | DPSC | Human | DPSC transplantation increased bone and periodontal ligament regeneration in mandibular bony defects |
| Zheng *et al.* (2009)^26^ | | Musculoskeletal | DPSC | Swine | DPSC transplantation increased bone regeneration in mandibular bony defects |
| Yang *et al.* (2010)^27^ | | Musculoskeletal | DPSC | Mouse | DPSC transplantation increased muscle regeneration in muscle defects |
| Honda *et al.* (2011)^28^ | | Musculoskeletal | DFSC | Rat | DFSC transplantation increased bone regeneration in calvaric defects |
| Pisciotta *et al.* (2012)^29^ | | Musculoskeletal | DPSC | Rat | DPSC transplantation increased bone regeneration in calvaric defects |
| Riccio *et al.* (2012)^30^ | | Musculoskeletal | DPSC | Rat | DPSC transplantation increased bone regeneration in calvaric defects |
| Giuliani *et al.* (2013)^31^ | | Musculoskeletal | DPSC | Human | DPSC transplantation increased bone regeneration in mandibular bony defects |
| Maraldi *et al.* (2013)^32^ | | Musculoskeletal | DPSC | Rat | DPSC transplantation increased bone regeneration in calvaric defects |
| Annibali *et al*. (2014)^33^ | | Musculoskeletal | DPSC | Rat | DPSC transplantation increased bone regeneration in calvaric defects |
| Fujii *et al.* (2018)^34^ | | Musculoskeletal | DPSC | Mouse | DPSC transplantation increased bone regeneration in calvaric defects |
| Liu *et al.* (2020)^35^ | | Musculoskeletal | ABMSC | Rabbit | DPSC transplantation increased bone regeneration in calvaric defects |
| Arthur *et al.* (2009)^36^ | | Neurodegenerative | DPSC | Avian | DPSC transplantation guided axon generation and neuroplasticity |
| Wang *et al.* (2010)^37^ | | Neurodegenerative | SHED | Mouse | SHED transplantation improved parkinsonian behaviour disorders |
| Matsubara *et al.* (2015)^38^ | | Neurodegenerative | SHED | Rat | SHED-conditioned media infusion increased functional recovery after spinal cord injury |
| Mita *et al.* (2015)^39^ | | Neurodegenerative | SHED | Mouse | SHED-conditioned media infusion increased cognitive function in Alzheimer’s model |
| Yamamoto *et al.* (2016)^40^ | | Neurodegenerative | DPSC | Rat | DPSC transplantation induced myelinated fibre regeneration |
| Nicola *et al.* (2017)^41^ | | Neurodegenerative | SHED | Rat | SHED transplantation increased functional recovery after spinal cord injury |
| Zhang *et al.* (2018)^42^ | | Neurodegenerative | SHED | Rat | SHED transplantation improved parkinsonian motor defects |
| Gomes *et al.* (2010)^43^ | | Ophthalmic | DPSC | Mouse | DPSC transplantation induces cornea regeneration in corneal defects |
| Syed-Picard *et al.* (2015)^44^ | | Ophthalmic | DPSC | Mouse | DPSC injection induces cornea regeneration in corneal defects |
| Ueda *et al.* (2010)^45^ | | Dermatological | SHED | Mouse | SHED-conditioned media infusion improved wound healing and reduced UV damage |
| Wakayama *et al.* (2015)^46^ | | Respiratory | SHED | Mouse | SHED-conditioned media infusion attenuated lung injury and improved survival rates |
| ABMSC: alveolar bone mesenchymal stem cell; DPSC: dental pulp stem cell; DFSC: dental follicle stem cell; GMSC: gingival mesenchymal stem cells; SCAP: stem cells of the apical papilla; SHED: stem cells from human exfoliated deciduous teeth | | | | | |
|  | |  |  |  |  |

Supplementary Table S1 References

1. Yamaza T, Kentaro A, Chen C, Liu Y, Shi Y, Gronthos S, et al. Immunomodulatory properties of stem cells from human exfoliated deciduous teeth. Stem Cell Res Ther. 2010;1(1):5.

2. Ishikawa J, Takahashi N, Matsumoto T, Yoshioka Y, Yamamoto N, Nishikawa M, et al. Factors secreted from dental pulp stem cells show multifaceted benefits for treating experimental rheumatoid arthritis. Bone. 2016;83:210-9.

3. Shimojima C, Takeuchi H, Jin S, Parajuli B, Hattori H, Suzumura A, et al. Conditioned Medium from the Stem Cells of Human Exfoliated Deciduous Teeth Ameliorates Experimental Autoimmune Encephalomyelitis. J Immunol. 2016;196(10):4164-71.

4. Gandia C, Armiñan A, García-Verdugo JM, Lledó E, Ruiz A, Miñana MD, et al. Human dental pulp stem cells improve left ventricular function, induce angiogenesis, and reduce infarct size in rats with acute myocardial infarction. Stem Cells. 2008;26(3):638-45.

5. Yamaguchi S, Shibata R, Yamamoto N, Nishikawa M, Hibi H, Tanigawa T, et al. Dental pulp-derived stem cell conditioned medium reduces cardiac injury following ischemia-reperfusion. Sci Rep. 2015;5:16295.

6. Chiu H-Y, Lin C-H, Hsu CY, Yu J, Hsieh C-H, Shyu W-C. IGF1R+ Dental Pulp Stem Cells Enhanced Neuroplasticity in Hypoxia-Ischemia Model. Molecular Neurobiology. 2017;54(10):8225-41.

7. Lee H-T, Chang H-T, Lee S, Lin C-H, Fan J-R, Lin S-Z, et al. Role of IGF1R+ MSCs in modulating neuroplasticity via CXCR4 cross-interaction. Scientific Reports. 2016;6(1):32595.

8. Miura M, Gronthos S, Zhao M, Lu B, Fisher LW, Robey PG, et al. SHED: stem cells from human exfoliated deciduous teeth. Proc Natl Acad Sci U S A. 2003;100(10):5807-12.

9. Seo B-M, Miura M, Gronthos S, Mark Bartold P, Batouli S, Brahim J, et al. Investigation of multipotent postnatal stem cells from human periodontal ligament. The Lancet. 2004;364(9429):149-55.

10. Sonoyama W, Liu Y, Fang D, Yamaza T, Seo B-M, Zhang C, et al. Mesenchymal Stem Cell-Mediated Functional Tooth Regeneration in Swine. PLOS ONE. 2006;1(1):e79.

11. Liu Y, Zheng Y, Ding G, Fang D, Zhang C, Bartold PM, et al. Periodontal ligament stem cell-mediated treatment for periodontitis in miniature swine. Stem Cells. 2008;26(4):1065-73.

12. Cordeiro MM, Dong Z, Kaneko T, Zhang Z, Miyazawa M, Shi S, et al. Dental Pulp Tissue Engineering with Stem Cells from Exfoliated Deciduous Teeth. Journal of Endodontics. 2008;34(8):962-9.

13. Ding G, Liu Y, Wang W, Wei F, Liu D, Fan Z, et al. Allogeneic periodontal ligament stem cell therapy for periodontitis in swine. Stem Cells. 2010;28(10):1829-38.

14. Huang GT, Yamaza T, Shea LD, Djouad F, Kuhn NZ, Tuan RS, et al. Stem/progenitor cell-mediated de novo regeneration of dental pulp with newly deposited continuous layer of dentin in an in vivo model. Tissue Eng Part A. 2010;16(2):605-15.

15. Alsanea R, Ravindran S, Fayad MI, Johnson BR, Wenckus CS, Hao J, et al. Biomimetic approach to perforation repair using dental pulp stem cells and dentin matrix protein 1. J Endod. 2011;37(8):1092-7.

16. Park JY, Jeon SH, Choung PH. Efficacy of periodontal stem cell transplantation in the treatment of advanced periodontitis. Cell Transplant. 2011;20(2):271-85.

17. Khorsand A, Eslaminejad MB, Arabsolghar M, Paknejad M, Ghaedi B, Rokn AR, et al. Autologous dental pulp stem cells in regeneration of defect created in canine periodontal tissue. J Oral Implantol. 2013;39(4):433-43.

18. Rosa V, Zhang Z, Grande RHM, Nör JE. Dental Pulp Tissue Engineering in Full-length Human Root Canals. Journal of Dental Research. 2013;92(11):970-5.

19. Yu X, Ge S, Chen S, Xu Q, Zhang J, Guo H, et al. Human gingiva-derived mesenchymal stromal cells contribute to periodontal regeneration in beagle dogs. Cells Tissues Organs. 2013;198(6):428-37.

20. Nakashima M, Iohara K. Mobilized dental pulp stem cells for pulp regeneration: initiation of clinical trial. J Endod. 2014 Apr;40(4 Suppl):S26-32.

21. Kanafi MM, Rajeshwari YB, Gupta S, Dadheech N, Nair PD, Gupta PK, et al. Transplantation of islet-like cell clusters derived from human dental pulp stem cells restores normoglycemia in diabetic mice. Cytotherapy. 2013;15(10):1228-36.

22. Cho YA, Noh K, Jue SS, Lee SY, Kim EC. Melatonin promotes hepatic differentiation of human dental pulp stem cells: clinical implications for the prevention of liver fibrosis. J Pineal Res. 2015;58(1):127-35.

23. Yamaza T, Alatas FS, Yuniartha R, Yamaza H, Fujiyoshi JK, Yanagi Y, et al. In vivo hepatogenic capacity and therapeutic potential of stem cells from human exfoliated deciduous teeth in liver fibrosis in mice. Stem Cell Research & Therapy. 2015;6(1):171.

24. de Mendonça Costa A, Bueno DF, Martins MT, Kerkis I, Kerkis A, Fanganiello RD, et al. Reconstruction of Large Cranial Defects in Nonimmunosuppressed Experimental Design With Human Dental Pulp Stem Cells. Journal of Craniofacial Surgery. 2008;19(1).

25. d'Aquino R, De Rosa A, Lanza V, Tirino V, Laino L, Graziano A, et al. Human mandible bone defect repair by the grafting of dental pulp stem/progenitor cells and collagen sponge biocomplexes. Eur Cell Mater. 2009;18:75-83.

26. Zheng Y, Liu Y, Zhang CM, Zhang HY, Li WH, Shi S, et al. Stem Cells from Deciduous Tooth Repair Mandibular Defect in Swine. Journal of Dental Research. 2009;88(3):249-54.

27. Yang R, Chen M, Lee CH, Yoon R, Lal S, Mao JJ. Clones of Ectopic Stem Cells in the Regeneration of Muscle Defects In Vivo. PLOS ONE. 2010;5(10):e13547.

28. Honda MJ, Imaizumi M, Suzuki H, Ohshima S, Tsuchiya S, Satomura K. Stem cells isolated from human dental follicles have osteogenic potential. Oral Surgery, Oral Medicine, Oral Pathology, Oral Radiology, and Endodontology. 2011;111(6):700-8.

29. Pisciotta A, Riccio M, Carnevale G, Beretti F, Gibellini L, Maraldi T, et al. Human Serum Promotes Osteogenic Differentiation of Human Dental Pulp Stem Cells In Vitro and In Vivo. PLOS ONE. 2012;7(11):e50542.

30. Riccio M, Maraldi T, Pisciotta A, La Sala GB, Ferrari A, Bruzzesi G, et al. Fibroin Scaffold Repairs Critical-Size Bone Defects In Vivo Supported by Human Amniotic Fluid and Dental Pulp Stem Cells. Tissue Engineering Part A. 2011;18(9-10):1006-13.

31. Giuliani A, Manescu A, Langer M, Rustichelli F, Desiderio V, Paino F, et al. Three Years After Transplants in Human Mandibles, Histological and In-Line Holotomography Revealed That Stem Cells Regenerated a Compact Rather Than a Spongy Bone: Biological and Clinical Implications. STEM CELLS Translational Medicine. 2013;2(4):316-24.

32. Maraldi T, Riccio M, Pisciotta A, Zavatti M, Carnevale G, Beretti F, et al. Human amniotic fluid-derived and dental pulp-derived stem cells seeded into collagen scaffold repair critical-size bone defects promoting vascularization. Stem Cell Research & Therapy. 2013;4(3):53.

33. Annibali S, Bellavia D, Ottolenghi L, Cicconetti A, Cristalli M, Quaranta R, et al. Micro-CT and PET analysis of bone regeneration induced by biodegradable scaffolds as carriers for dental pulp stem cells in a rat model of calvarial “critical size” defect: Preliminary data. Journal of biomedical materials research Part B, Applied biomaterials. 2014;102.

34. Fujii Y, Kawase-Koga Y, Hojo H, Yano F, Sato M, Chung U-i, et al. Bone regeneration by human dental pulp stem cells using a helioxanthin derivative and cell-sheet technology. Stem Cell Research & Therapy. 2018;9(1):24.

35. Liu Y, Wang H, Dou H, Tian B, Li L, Jin L, et al. Bone regeneration capacities of alveolar bone mesenchymal stem cells sheet in rabbit calvarial bone defect. J Tissue Eng. 2020;11:2041731420930379.

36. Arthur A, Shi S, Zannettino AC, Fujii N, Gronthos S, Koblar SA. Implanted adult human dental pulp stem cells induce endogenous axon guidance. Stem Cells. 2009;27(9):2229-37.

37. Wang J, Wang X, Sun Z, Wang X, Yang H, Shi S, et al. Stem cells from human-exfoliated deciduous teeth can differentiate into dopaminergic neuron-like cells. Stem Cells Dev. 2010;19(9):1375-83.

38. Matsubara K, Matsushita Y, Sakai K, Kano F, Kondo M, Noda M, et al. Secreted ectodomain of sialic acid-binding Ig-like lectin-9 and monocyte chemoattractant protein-1 promote recovery after rat spinal cord injury by altering macrophage polarity. J Neurosci. 2015;35(6):2452-64.

39. Mita T, Furukawa-Hibi Y, Takeuchi H, Hattori H, Yamada K, Hibi H, et al. Conditioned medium from the stem cells of human dental pulp improves cognitive function in a mouse model of Alzheimer's disease. Behav Brain Res. 2015;293:189-97.

40. Yamamoto T, Osako Y, Ito M, Murakami M, Hayashi Y, Horibe H, et al. Trophic Effects of Dental Pulp Stem Cells on Schwann Cells in Peripheral Nerve Regeneration. Cell Transplant. 2016;25(1):183-93.

41. Nicola FDC, Marques MR, Odorcyk F, Arcego DM, Petenuzzo L, Aristimunha D, et al. Neuroprotector effect of stem cells from human exfoliated deciduous teeth transplanted after traumatic spinal cord injury involves inhibition of early neuronal apoptosis. Brain Res. 2017;1663:95-105.

42. Zhang N, Lu X, Wu S, Li X, Duan J, Chen C, et al. Intrastriatal transplantation of stem cells from human exfoliated deciduous teeth reduces motor defects in Parkinsonian rats. Cytotherapy. 2018;20(5):670-86.

43. Gomes JA, Geraldes Monteiro B, Melo GB, Smith RL, Cavenaghi Pereira da Silva M, Lizier NF, et al. Corneal reconstruction with tissue-engineered cell sheets composed of human immature dental pulp stem cells. Invest Ophthalmol Vis Sci. 2010;51(3):1408-14.

44. Syed-Picard FN, Du Y, Lathrop KL, Mann MM, Funderburgh ML, Funderburgh JL. Dental pulp stem cells: a new cellular resource for corneal stromal regeneration. Stem Cells Transl Med. 2015;4(3):276-85.

45. Ueda M, Nishino Y. Cell-based cytokine therapy for skin rejuvenation. J Craniofac Surg. 2010;21(6):1861-6.

46. Wakayama H, Hashimoto N, Matsushita Y, Matsubara K, Yamamoto N, Hasegawa Y, et al. Factors secreted from dental pulp stem cells show multifaceted benefits for treating acute lung injury in mice. Cytotherapy. 2015;17(8):1119-29.
